# Integrin-Blocking Antibodies Delay Keratinocyte Re-Epithelialization in a Human Three-Dimensional Wound Healing Model

**DOI:** 10.1371/journal.pone.0010528

**Published:** 2010-05-21

**Authors:** Christophe Egles, Heather A. Huet, Furkan Dogan, Sam Cho, Shumin Dong, Avi Smith, Elana B. Knight, Karen R. McLachlan, Jonathan A. Garlick

**Affiliations:** 1 Division of Cancer Biology and Tissue Engineering, Department of Oral and Maxillofacial Pathology, School of Dental Medicine, Tufts University, Boston, Massachusetts, United States of America; 2 Discovery Oncobiology, Biogen Idec, Cambridge, Massachusetts, United States of America; 3 Translational Oncobiology, Biogen Idec, San Diego, California, United States of America; Massachusetts Institute of Technology, United States of America

## Abstract

The α6β4 integrin plays a significant role in tumor growth, angiogenesis and metastasis through modulation of growth factor signaling, and is a potentially important therapeutic target. However, α6β4-mediated cell-matrix adhesion is critical in normal keratinocyte attachment, signaling and anchorage to the basement membrane through its interaction with laminin-5, raising potential risks for targeted therapy. Bioengineered Human Skin Equivalent (HSE), which have been shown to mimic their normal and wounded counterparts, have been used here to investigate the consequences of targeting β4 to establish toxic effects on normal tissue homeostasis and epithelial wound repair. We tested two antibodies directed to different β4 epitopes, one adhesion-blocking (ASC-8) and one non-adhesion blocking (ASC-3), and determined that these antibodies were appropriately localized to the basal surface of keratinocytes at the basement membrane interface where β4 is expressed. While normal tissue architecture was not altered, ASC-8 induced a sub-basal split at the basement membrane in non-wounded tissue. In addition, wound closure was significantly inhibited by ASC-8, but not by ASC-3, as the epithelial tongue only covered 40 percent of the wound area at 120 hours post-wounding. These results demonstrate β4 adhesion-blocking antibodies may have adverse effects on normal tissue, whereas antibodies directed to other epitopes may provide safer alternatives for therapy. Taken together, we conclude that these three-dimensional tissue models provide a biologically relevant platform to identify toxic effects induced by candidate therapeutics, which will allow generation of findings that are more predictive of *in vivo* responses early in the drug development process.

## Introduction

Integrins are heterodimeric cell surface adhesion receptors expressed on most cells throughout the body where they mediate cell-cell and cell-extracellular matrix interactions [1]. They consist of α and β subunits that associate in various combinations to form at least 25 receptors. Each αβ combination possesses specific binding and signaling properties. Integrins are receptors for extracellular matrices that transmit mechanical and biochemical signals to regulate cellular functions including survival, proliferation, motility, transcription and protein translation [2], [3]. In normal tissues, α6β4 plays a role in the maintenance of epithelial integrity, particularly in the epidermis where as a component of the hemidesmosome complex, it serves to anchor basal keratinocytes to the underlying basement membrane through its interaction with laminin-5 or laminin-322 according to the new laminin nomenclature [4] and other proteins of the complex [5]. These interactions are destabilized when hemidesmosome disassembly is required, for instance, to allow keratinocyte migration during wound healing, a process regulated through cooperation between β4 and growth factor mediated signaling [6].

In the last decade, experimental evidence has emerged that integrins are involved in cancer growth, angiogenesis and metastasis, and several antibodies targeting integrins are being clinically evaluated as treatments for cancer. Integrins influence tumor progression by modifying various intracellular signaling pathways. α6β4, in particular, has been shown to cooperate in growth factor mediated signaling [7]. Integrin α6β4 enhances pro-tumor functions, such as migration, invasion, and resistance to apoptotic stimuli [8]. In *in vivo* animal models, α6β4-signaling was found to promote the onset of pathologic angiogenesis and tumorigenesis [9], [10]. In these experiments, the growth of xenograft tumor could be inhibited with a β4 antibody. Furthermore, α6β4 expression is increased in several types of invasive and metastatic human carcinomas including breast, colon, thyroid, gastric, bladder and squamous cell carcinomas [8], [11]. Because of this putative role in cancer, we and others have proposed β4 as a potentially important therapeutic target that may be amenable to an antibody blocking approach. However, the consequences of targeting α6β4 integrin with an antibody on normal tissue homeostasis and repair processes haven't been yet studied.

In order to assess potential secondary effects of blocking α6β4 on human stratified squamous epithelium, we utilized bioengineered three-dimensional tissues that mimic human skin known as human skin equivalents (HSEs). We have previously described the development of HSEs, in which epidermal cells are grown at an air–liquid interface on a connective tissue substrate harboring viable fibroblasts [12]. In these three-dimensional tissues, HSEs express basement membrane components, such as β4 and laminin-5, recapitulating the organization of basement membrane in human stratified squamous epithelium. Moreover, we adapted HSEs to study wound repair in a manner that simulates the chronology of events that occur during re-epithelialization in human skin [13], [14]. These HSEs enable direct determination of phenotypic response of a wounded epithelium including cell proliferation, migration, differentiation, growth-factor response, and protease expression of epithelial and stromal cells. Importantly, these tissues now serve as platforms for the rapid screening of the potential efficacy or toxicity of human therapeutics.

We previously demonstrated that an antibody (ASC-3) directed to β4 could prevent anchorage-independent growth of breast tumor cells in preclinical assays [15]. The activity and epitope of this antibody was distinguishable from an adhesion blocking antibody (ASC-8) that disrupts β4 interactions with laminin-5, but does not affect growth in soft agar. We used three-dimensional tissues as a platform to test the consequences of targeting β4 with these antibodies, which are specific to human β4, first in a static three-dimensional tissue model of non-wounded human epithelium and second in a dynamic three-dimensional tissue model of wound healing. When these antibodies were added to the culture medium of HSEs, they adhered to the basement membrane zone at the interface between the connective tissue and basal keratinocytes, demonstrating appropriate localization of the antibody to areas expressing the β4 subunit. However, while ASC-8 caused detachment of the epithelium from the underlying stroma and significantly blocked wound reepithelialization, ASC-3 neither blocked adhesion nor disrupted wound closure. This was partially explained by the finding that ASC-8, but not ASC-3, significantly decreased the migration rate of human keratinocytes in two-dimensional cultures and was associated with a reduction in cellular proliferation in the three-dimensional wound healing model. These results indicate that therapeutic targeting with β4 blocking antibodies that disrupt cell adhesion to laminin-5 could have potentially adverse side effects on normal tissue homeostasis and wound repair. Furthermore, this study demonstrates the utility of human, three-dimensional tissue platforms to screen the function of antibodies directed to different epitopes. These platforms show promise in efficiently and economically enabling determination of the safety profile of candidate therapeutic agents.

## Materials and Methods

All the cells used in this study have been isolated and published previously [12]–[14], [16].

### Adhesion assays to determine ASC-3 and ASC-8 binding to laminin-5

For cellular adhesion assays, Human Foreskin Keratinocytes and an immortalized keratinocyte cell line (HACAT) were first grown in serum-free medium (Dulbecco's Modified Eagle Medium, Invitrogen) for 24 hours. Cells were harvested using cell dissociation buffer (Invitrogen) and resuspended at 1106 cells/ml in Leibovitz L-15 medium (Invitrogen). For antibody treatments, 10 µg/ml of ASC-3 or ASC-8 (Chemicon) were added to the resuspended cells, and incubated for 1 hr on ice. Cells were then plated on 96-well plate that were precoated with 10 µg/ml rat laminin-5 (Millipore), or a mixture of human laminins (Sigma), at 1×10^5^ cells per well and incubated for 1 hr at 4°C. After washing the plates with Leibovitz L-15 medium, adherent cells were quantified using Cell Titer Glo cell viability assay (Promega) and luminescence reading on a SpectraMax M5 plate reader.

### Flow Cytometry

Cells were harvested using cell dissociation buffer (Invitrogen), washed and resuspended in FACS wash buffer (PBS) containing 10% normal goat serum (Invitrogen), 0.2% Bovine Serum Albumin (Sigma), 0.1% NaN_3_ (Sigma) at a concentration of 1×10^6^ cells/well. Antibodies were diluted in FACS wash buffer and incubated with the cells for 1 hr on ice, followed by washing and incubation with a phycoerythrin-conjugated goat anti-mouse secondary antibody for 1 hr on ice. Cells were washed again and resuspended in FACS wash buffer containing 1 ug/ml propidium iodide (PI) (Molecular Probes, Eugene, OR). Viable cells were measured using a FACSArray bioanalyzer (BD Biosciences), and mean fluorescence intensity was graphed using GraphPad PRISM (GraphPad Software, San Diego, CA).

### Scratch wound healing assay in two-dimensional culture

Keratinocytes were grown to confluence and wounded with a sterile 0.1- to 10-µl pipette tip. Six perpendicular linear scratches were generated and the repopulation of the wound gaps created was photographed. Differences in cell migration were calculated by measuring repopulation of the scratch wound after 12 hr when compared to the original wound edge (time zero). For each condition, results were expressed as a percent wound closure when compared to control wounds, (100% closure). Two independent experiments with a minimum of three observations for each condition were analyzed.

### Fabrication of HSE to generate static (non-wounded) and dynamic (wounded) three-dimensional model

Skin equivalent cultures were generated as previously described [17]. Briefly, to construct the collagen matrix, HDF cells were added to neutralized Type I Collagen (Organogenesis, Canton, MA) to a final concentration of 2.5×10^4^ cells/ml; 3 ml of this mixture was added to each 35 mm well insert of a 6-well plate and incubated for 7 days in media containing DMEM and 10% fetal calf serum until the collagen gel showed no further shrinkage. At this time, of 5×10^5^ NHKs were plated directly on a raised, mesa-like area in the center of the contracted collagen gel. Cultures were submerged in low calcium epidermal growth media for 2 days, submerged in normal calcium epidermal growth media for 2 days and raised to the air-liquid interface by feeding from below at 37°C in 7.5% CO2. The cultures were wounded 7 days after keratinocytes were seeded onto the collagen matrix. An additional collagen matrix was fabricated and used as the substrate onto which the wounded skin equivalents were transferred. To generate wounds, skin equivalent cultures were removed from the insert membrane, and a 1.2-cm incision that penetrated the epidermis and collagen matrix was created by using a 4 cm punch (Delasco, Inc.) resulting in an elliptical circular wound. The wounded tissue was transferred onto the second, previously fabricated, collagen matrix. Wounded cultures were maintained at the air–liquid interface for 24, 48, and 72 hr at 37°C in 7.5% CO2 to monitor reepithelialization.

### Morphologic analysis and Immunofluorescence staining

For light microscopy, skin equivalent cultures were fixed in 4% neutral buffered formalin, embedded in paraffin, and serially sectioned at 8 µm. Sections were stained with hematoxylin and eosin, and visualized using a Nikon Eclipse 80i microscope equipped with Diagnostic Instruments SPOT-RT Camera. For epifluorescence microscopy, tissues were harvested and frozen in embedding medium (Triangle Biomedical) after being placed in 2 M sucrose overnight at 4°C. Frozen tissues were serially sectioned at 8 µm and stained with secondary antibodies (Boehringer-Mannheim) and counterstained with DAPI (4,6-diamidino-2-phenylindole) (Vector).

## Results

### ASC-8, but not ASC-3, blocks interaction between β4 and laminin-5 in human keratinocytes

**Figure 1:**
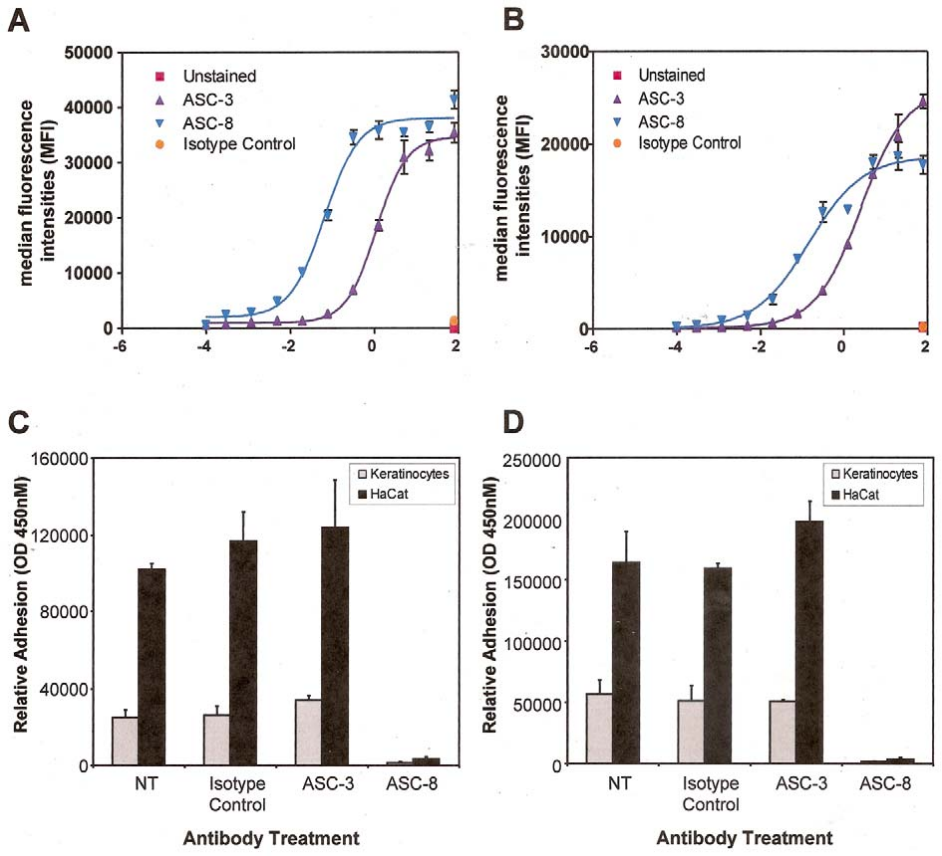
Characterization of β4-specific antibody effects on human keratinocytes. The binding of control or β4-specific antibodies ASC-3 and ASC-8 to normal human keratinocytes (A) and HaCaT cells (B) was measured by flow cytometry. To determine the effects on adhesion, serum starved cells were either left untreated (NT), or incubated with control or β4-specific antibodies prior to plating on rat laminin-5 (C) and a mixture of human laminins (D) Relative adhesion was measured using a luciferase based luminescent viability assay. Experiments were performed twice in triplicate and results shown are from one representative experiment.

Expression of α6β4 was detected in the human adult keratinocytes (NHK) as well as in HaCaT cells, a spontaneously immortalized human keratinocyte cell line by flow cytometry. The titration of α6β4 using the FACS technique with both antibodies (ASC-3 and ASC-8) showed strong binding to these cells, demonstrating the constitutive expression of α6β4 in both cell types. The measured EC50 on these two types of keratinocytes was higher for HaCaT than NHK for ASC-3 (NHK: 1.9 nM: HaCaT: 2.6 nM) as well as for ASC-8 (NHK: 0.065 nM; HaCaT: 0.15 nM). (Figure 1a and b)

The β4-specific antibody ASC-8 has previously been shown to block adhesion to laminin in cells expressing endogenous α6β4 [18]. We therefore examined whether the same response could be observed for α6β4-expressing keratinocytes. Preincubation of ASC-8 with either NHK or HaCaT prior to incubation of cells on human laminin-1 (Figure 1c) or laminin-5-coated plates (Figure 1d) resulted in complete blockade of endogenous α6β4-laminin interactions. Interestingly, preincubation with the ASC-3 antibody had no inhibitory effect on a6b4-laminin interactions similar to the isotype control. These observations further confirmed the presence of α6β4 integrins on both keratinocyte cell types. Moreover, this demonstrated that the epitope recognized by ASC-8 plays a critical role in mediating the adhesive interactions between the β4 subunit with laminin-5, whereas the epitope recognized by ASC-3 seems to be irrelevant for cell adhesion.

### Blocking inhibition of cell migration in a two-dimensional scratch assay contains the functional blocking capacity of ASC-8 antibody

**Figure 2:**
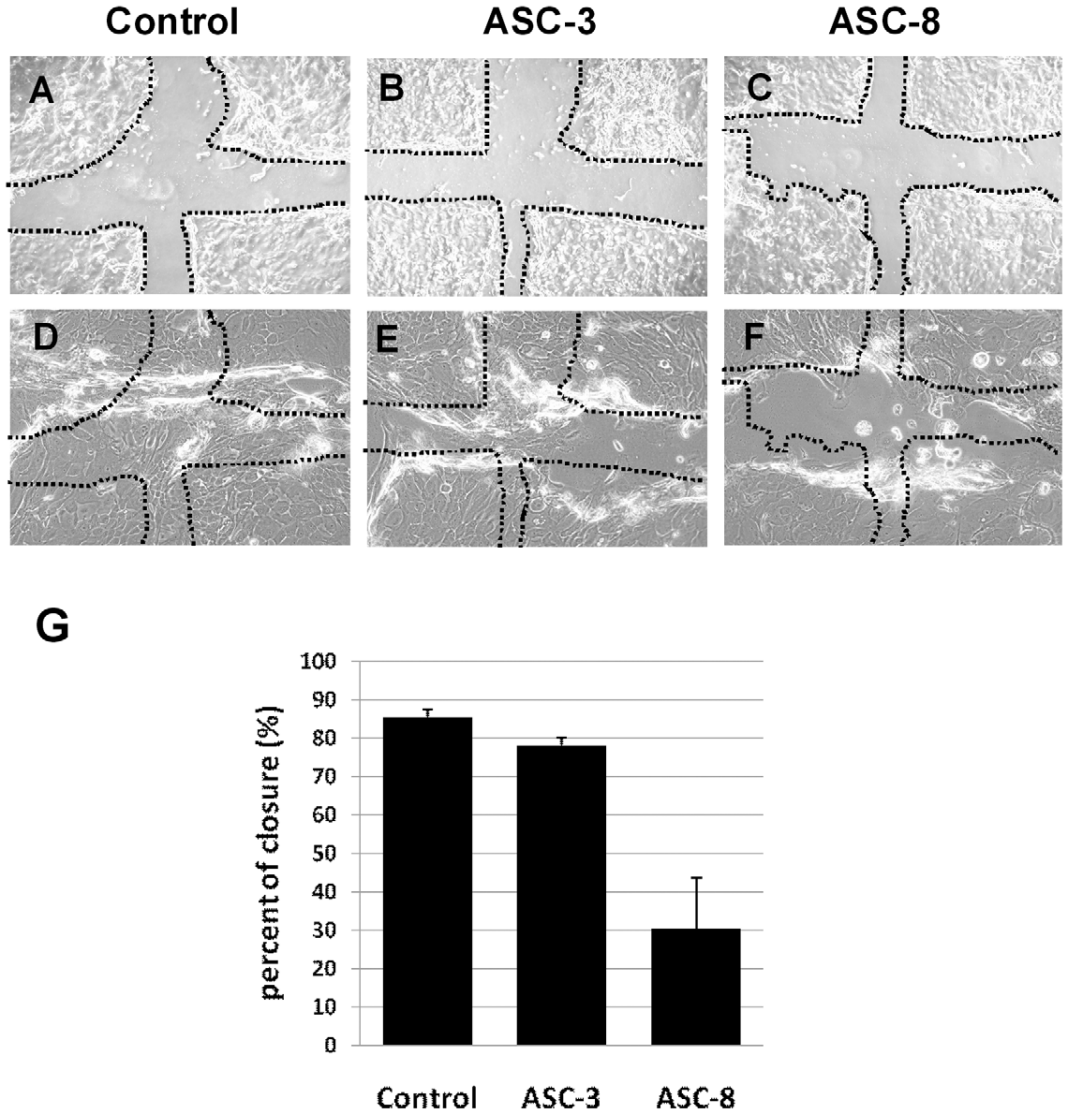
Immunoblocking of integrins decreases the rate of cell motility. The initial scratch wound was carried out on uncoated culture dishes with confluent keratinocyte cultures (A), and on keratinocyte cultures treated with ASC-3 (B) and ASC-8 (C). Panels D–F illustrate repopulation of the wound surface after 24 hours by keratinocytes without the presence of antibodies (D), or in the presence of ASC-3 (E), or ASC-8 (F). Cells that migrated into the wound gap were calculated for each condition and the differences were expressed as a percentage of wound closure of NHK cell culture (G). Each bar represents the mean and the standard deviation of triplicate determinations in two separate experiments.

In order to assess possible different effects of the two β4 antibodies on keratinocyte migration, we performed scrape wound assays in which confluent keratinocyte monolayers, cultured in uncoated plates, were mechanically disrupted to create an area devoid of cells (Figure 2a–c). Wounded cultures were then treated with one of the antibody at a saturating antibody concentration of 25 µg/ml. When the rate of cell repopulation of the scratch was measured, both untreated keratinocytes (Figure 2d) as well as ASC-3 treated cultures (Figure 2e) were found to repopulate the wound gap 24 hours after wounding. In contrast, ASC-8-treated cultures did not repopulate the wound gap, as only a few cells were found to migrate into the wound after 12 hr (Figure 2f). As summarized in Figure 2g, quantitative analysis of scrape wounds revealed a significant decrease in the rate of cell migration in ASC-8 treated cells (29%±10.9%) in comparison to ASC-3 treated (79%±1.3) and control untreated cultures (84%±2.3). These results confirmed that this inhibition of keratinocyte migration was dependent on the epitopes of the blocking antibody, and that laminin-5-β4 interactions are necessary for keratinocyte migration.

### Antibody blocking of integrin subunit β4 in a non-wounded static three-dimensional human skin equivalent

**Figure 3:**
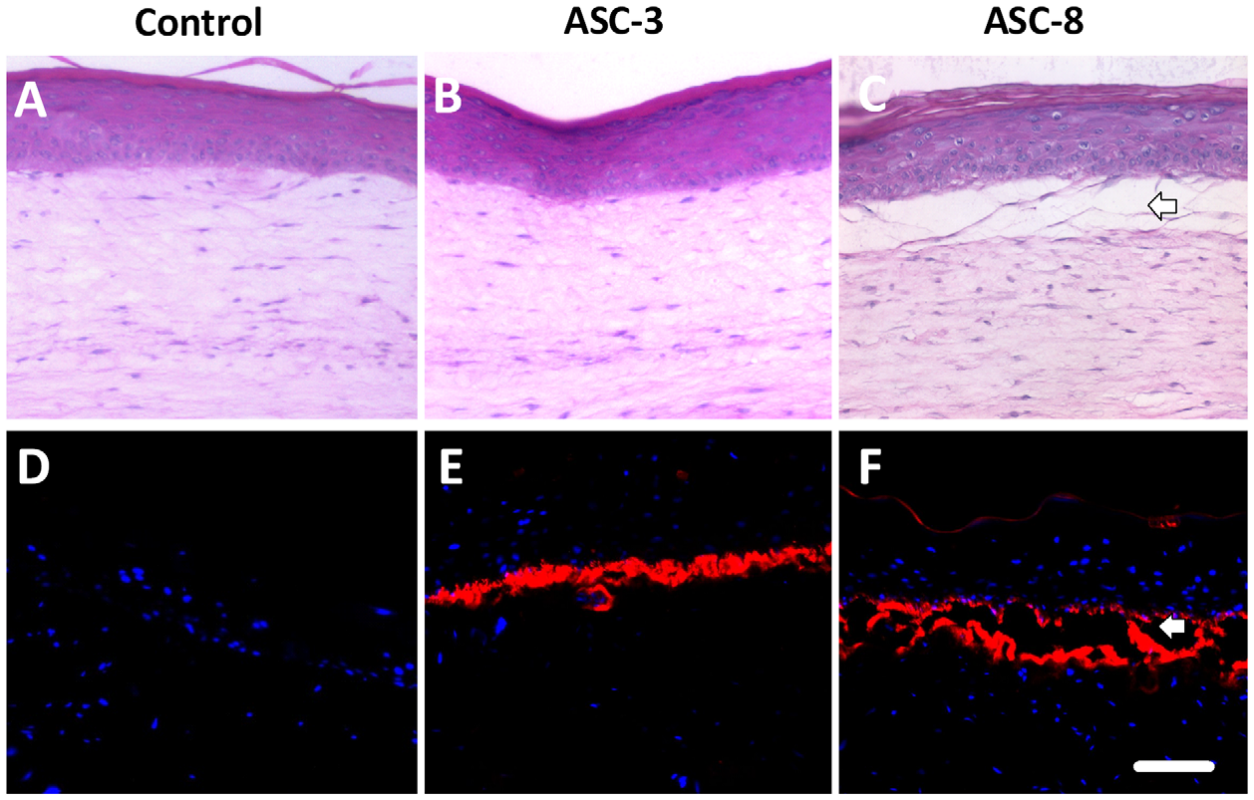
Immunodetection of the blocking antibodies in the Human Skin Equivalents. H and E staining of tissues revealed no differences in tissue architecture or numbers of cell layers between the control (A) and the ASC-3 (B) or ASC-8 (C) treated HSEs. No antibody was detected in the control tissues not exposed to antibodies. (D). While both ASC-3 (E) and ASC-8 (F) antibodies detected inside the tissue at the junction between dermis and epidermis, indicating specific localization by target along the basement membrane interface (C and F) ASC-8 is associated with a large disruption of the tissue integrity between the dermis and epidermis (F, white arrow).

**Figure 4:**
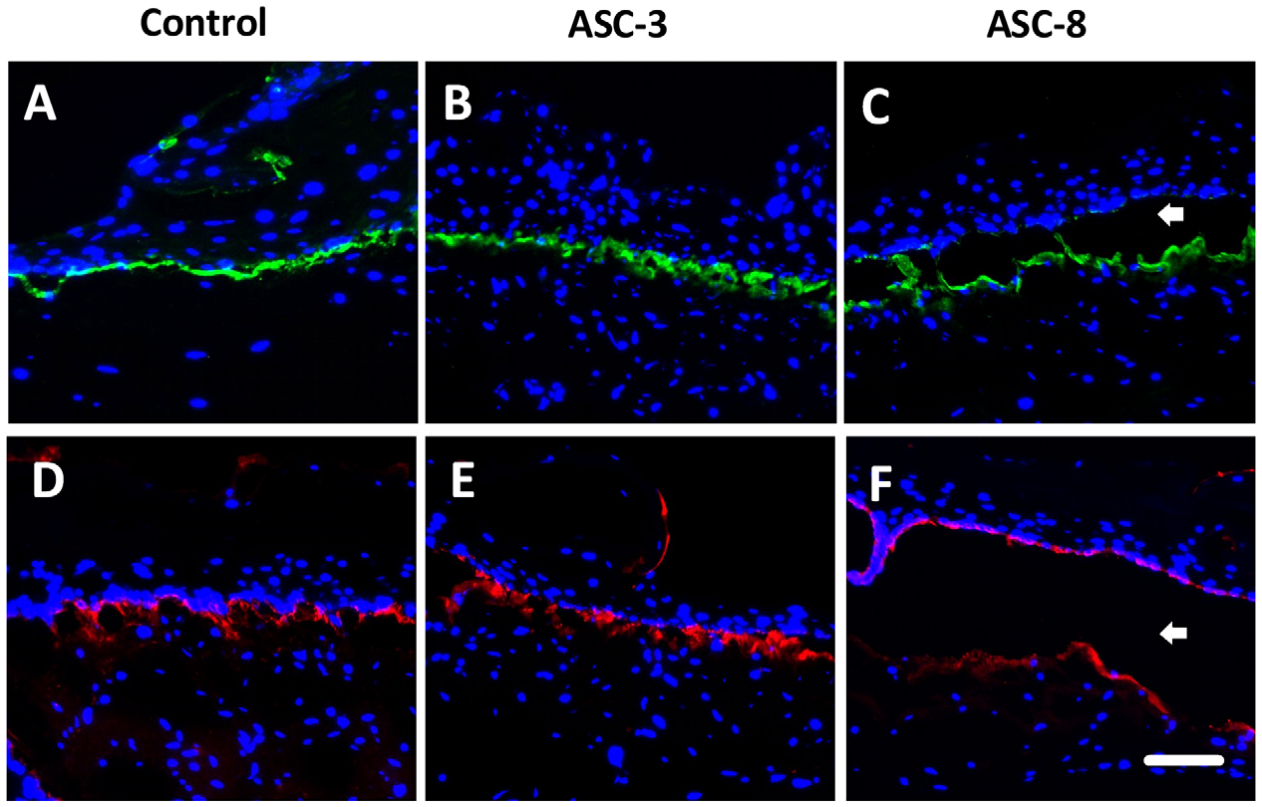
Immunofluorescent detection of the Basement Membrane Proteins. The immunodetection of alpha6 integrin shows no difference between the control (A) and the presence of ASC-3 and ASC-8 antibodies in the medium (B and C). The same observation can be made for laminin-5 (D, E and F). Note that in all cases (C),(F) ASC-8 is associated with large disruption of the tissue between dermis and epidermis (white arrow).

In order to test if the blocking of laminin-5-β4 interactions could lead to morphological differences in the tissues, the HSEs were incubated with ASC-3 and ASC-8 in the culture medium. No morphological differences could be detected upon Hematoxilin and Eosin staining when tissues were incubated with ASC-3 or ASC-8. (Figure 3a–c). To assess the presence of ASC-3 and ASC-8 in the HSE, the two antibodies were visualized using a secondary antibody directed against mouse Fc coupled to phycoerythrin (Fig. 3 red staining). No signal could be detected in the control HSE lacking antibodies in the culture medium (Fig. 3d) while both ASC-3 and ASC-8-treated cultures (Fig. 3e and f) demonstrated antibody localization to the basement membrane interface. The presence of ASC-3 antibodies was localized to an intact interface between dermis and epidermis, as seen by the linear stain along the basement membrane interface. In contrast, the adhesion-blocking antibody, ASC-8, caused disruption of this interface as evidenced by a split seen just below the basal keratinocytes between epidermis and dermis. Laminin-5 and α6 integrin serve as markers of the degree of organization of the epithelial–stromal interface. The linear interface deposition of α6 integrin (Figure 4a–c) and laminin-5 (Figure 4d–f) polarized along the basement membrane were found in the skin equivalents, in spite of the tissue disruption. In light of our previous findings that the linear, co-localization of these proteins was linked to basement membrane assembly [12], [19], this distribution is an indication of the unperturbed organization of basement membrane in ASC-3 treated tissues and abrogation of basement membrane assembly in ASC-8 treated tissues.

### ASC-8-antibody blocks re-epithelialization in a dynamic HSE model of wound repair

**Figure 5:**
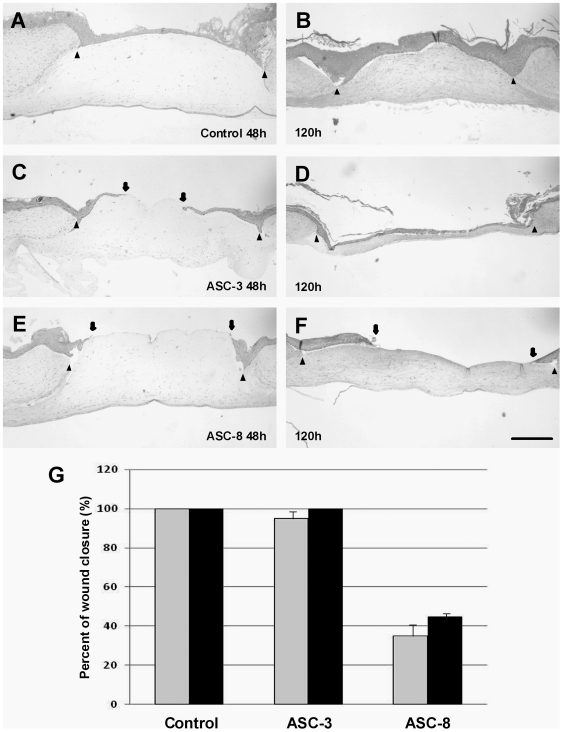
Hematoxylin and Eosin staining of *in vitro* wound after 48 and 120 hours. Compared to control wounds at 48 h (A) or 120 h (B) after wounding, with antibody ASC-3, epithelial tongue has covered more than 90% at 48 hour (between arrows) (C) and is totally close after 120 hours (D). However, ASC-8 completely inhibited wound closure (E). Even after 120 hours no wound closure can be observed (F). (E) Percentage of wound closure (grey bars: closure after 48 hours; black bars: closure after 120 hours). Results from two separate experiments are presented as mean±SD. Bar: 500 µm.

We next studied the effects of targeting β4 in wounded HSEs, which faithfully recapitulate the keratinocyte migration and proliferation seen in cutaneous healing of wounds *in vivo*. We used our previously-developed three-dimensional HSE model of cutaneous wound repair [13] to determine if blocking β4 integrin with ASC-3 or ASC-8 could also differentially alter the rate of re-epithelialization of wounded HSEs. After 24 hours, epithelial tongues were seen at the edge of the wound margin, indicating re-epithelialization was initiated at this time. By 48 hours post-wounding, the wound bed was completely covered by keratinocytes and tissue integrity was restored. To determine if the rate of wound healing differed in the presence of different antibodies, the distance separating the edge two epithelial tongues was measured and normalized to the location of the initial wound margins. The rate of wound closure was assessed by comparing the degree of re-epithelialization from the wound edge at 48 (Figure 5a,c and e) and 120 hours (Figure 5b, d and f) after wounding. HSEs wounded in the presence of ASC-3 (Figure 5c,d) showed a rate of wound closure similar to control tissues not exposed to antibody (Figure 5a,b). In contrast, the presence of ASC-8 significantly reduced the rate of re-epithelialization after 48 hours (Figure 5e)(Control = 100%, ASC-3 = 96±3.2% and ASC-8 = 36±5.4%) as well as after 120 hours (Figure 5f)(Control = 100%, ASC-3 = 100% and ASC-8 = 42±1.5%). These results indicate that disruption of the interactions between laminin-5 and β4 inhibit wound re-epithelialization when compared to non-adhesion blocking, anti-β4 antibodies.

## Discussion

Integrin α6β4 has been shown to be important for normal tissue homeostasis through its role in anchoring cells to the basement membrane and cell signaling. α6β4 is also an important therapeutic target in human cancer, with established roles in cancer cell adhesion, migration, and angiogenesis. The dysregulation of this integrin is known to promote tumor growth by altering the proliferation and migration of epithelial tumor cells that is mediated by growth factor receptor signaling. Several studies have linked α6β4 to cooperative signaling with growth factor receptors, particularly Epidermal growth factor receptor (EGFR), erythroblastosis oncogene B (ErbB2), and c-Met, through the Phosphoinositide 3-kinase pathway to facilitate tumorigenesis [9], [20]–[23]. Mice deficient in the long cytoplasmic domain of β4, while maintaining functional epithelial adhesion, show drastically reduced tumor size associated with less vascularization, implicating β4 in tumor angiogenesis [24]. These studies and others suggest α6β4 targeted therapy could have broad implications for cancer therapy, particularly in combination with other targeted agents in biological pathways, such as EGFR inhibitors, Herceptin™ (targeting ErbB2), or Avastin™ (targeting Vascular endothelial growth factor). Therefore, α6β4 is an attractive target for antibody therapy to treat epithelial cancers.

In the current study, we have addressed the safety concerns of targeting the β4 integrin by using Human Skin Equivalents (HSEs) as a screening platform to determine if antibodies that target two different epitopes in the β4 subunit can disrupt the structure or function of HSEs. Because α6β4 is involved in normal physiological processes, such as tissue homeostasis and wound healing, it was important to use an *in vitro* tissue model that could recapitulate these processes in order to test β4 inhibitors in the early drug discovery process. In addition, these antibodies are specific for human β4 subunit, and thus require a fully human tissue model for making safety assessments. Therefore, we chose the well described HSE tissue model, which recapitulates human skin and wound healing, to test antibodies directed at β4. Our previous results [15] demonstrated that the adhesion-blocking antibody ASC-8, had no effect on anchorage-independent growth of tumor cells, but a non-adhesion blocking antibody, ASC-3, did inhibit this β4 mediated function. We have extended these studies to show that ASC-8 disrupts the attachment of basal keratinocytes at the basement membrane interface and has a profound effect on wound closure by blocking migration of keratinocytes in the epithelial tongue of wounded HSEs.

In our study, both laminin-5 and integrin subunit α6 were distributed along the basement membrane interface in non-treated and ASC-3-treated samples. Tissues treated with ASC-8 showed a split beneath the non-wounded epithelium in a pattern in which laminin-5 was localized both to the epidermal and dermal side of the basement membrane interface. It is likely that the split seen in normal HSEs is due to the blocking of β4 integrin by ASC-8. Our additional results in two-dimensional scratch wound assays, demonstrating the profound blocking effect of ASC-8, may also be due to the ability of the blocking antibody ASC-8 to inhibit keratinocyte migration. Therefore, antibodies to distinct epitopes of β4 integrin can distinguish between its adhesive functions that were blocked with ASC-8 and functions not linked to wound healing or cell migration such as those seen with ASC-3. The results in the HSE demonstrate that the blocking the interaction of β4 with laminin-5 could have deleterious consequences. This sub-basal split seen under the stratum basale was similar to the pattern previously reported from the skin of patients with junctional epidermolysis bullosa, in which mutation of the β4 subunit resulted in separation of the epithelium at the basement membrane interface [25], [26]. Interestingly, the non-adhesion-blocking antibody, ASC-3, demonstrated anti-tumor effects *in vitro* in our prior studies [15], supporting the conclusion that the tumorigenic properties of β4 can be targeted independently of the adhesive properties to laminins.

Our results have broader implications to the drug discovery process because they also demonstrate the utility of three-dimensional tissue technology to assess the behavior of targeted therapy in a fully human tissue setting. A greater number of therapies targeting the tumor microenvironment, such as α6β4, are being investigated by the pharmaceutical industry, so understanding the behavior of these drugs in complex biological systems is information that can not be garnered from traditional cell culture models [27], [28]. Moreover, it is often difficult to test these new drugs using human antibodies in mouse models due to lack of cross-reactivity with the murine target. Testing such antibody candidates in three-dimensional human tissue models allows predictions of the safety of several candidate molecules long before preclinical testing in non-human primates or in human clinical situations [29]. Until recently, experimental paradigms have been based primarily on two-dimensional, monolayer culture systems. However, the ability of these approaches to simulate biological processes in human tissues has been limited, as it is known that biologically-meaningful signaling pathways function optimally when cells are spatially organized in three-dimensional tissues, but are uncoupled and lost in rudimentary two-dimensional culture systems. As we have shown in the current study, these three-dimensional tissue platforms are now able to yield biologically-relevant information that can be adapted to serve as human, “pre-clinical” or “surrogate” tissues that act as a translational modality to provide more meaningful correlations between *in vitro* screening assays for toxicity and efficacy and *in vivo* tissue outcomes in human clinical trials.
